# Early-onset low rectal adenocarcinoma with a pMMR/MSS phenotype in a patient with an analytically confirmed germline pathogenic PMS2 variant: a case report

**DOI:** 10.3389/fmed.2026.1945282

**Published:** 2026-10-30

**Authors:** Ying Li, Heming Ma, Fuxue Huang, Zhuo Wang, Meichen Gu, Pengyu Chang

**Affiliations:** 1 Department of Radiation Oncology & Therapy, The First Hospital of Jilin University, Changchun, China; 2 Department of Gastroenterology, The First Hospital of Jilin University, Changchun, China

**Keywords:** early-onset rectal cancer, germline testing, Lynch syndrome, microsatellite instability, mismatch repair

## Abstract

Mismatch repair (MMR) protein immunohistochemistry (IHC) and microsatellite instability (MSI) testing are central to colorectal cancer treatment stratification and hereditary-risk assessment, but a pMMR/MSS tumour phenotype does not exclude a germline predisposition in selected patients. We report a 41-year-old woman with low rectal adenocarcinoma whose pretreatment biopsy and resection specimen retained MLH1, PMS2, MSH2 and MSH6 expression and whose tumour was microsatellite stable (MSS) with low tumour mutational burden (TMB-L). Tumour-tissue sequencing initially identified the PMS2 frameshift variant NM_000535.7:c.1577del (p.Asp526AlafsTer69; GRCh37/hg19 chr7:6026819delT). A subsequent orthogonal assay using PMS2-specific three-step nested genomic PCR, TA cloning and Sanger sequencing of peripheral-blood DNA confirmed that the variant mapped to PMS2 and supported its germline origin; the clinical laboratory classified the variant as pathogenic using an ACMG/AMP-based interpretation. No dedicated analyses of tumoural PMS2 somatic second-hit events, loss of heterozygosity, copy-number alterations or promoter methylation were performed; therefore, biallelic PMS2 inactivation was not established. Postoperative circulating tumour DNA molecular residual disease was positive, and a left supraclavicular lymph-node biopsy subsequently confirmed metastatic adenocarcinoma consistent with colorectal origin. This case illustrates that pMMR/MSS findings should not be used in isolation to exclude hereditary risk in patients with early-onset disease or other clinical indications for germline assessment. These findings support selective germline assessment in patients with early-onset or otherwise high-risk pMMR/MSS colorectal cancer, but do not justify universal testing or establish that the tumour was caused by PMS2-associated Lynch syndrome.

## Introduction

Mismatch repair (MMR) protein immunohistochemistry (IHC) and microsatellite instability (MSI) testing are standard molecular assays in colorectal cancer management and are included in ASCP/CAP molecular biomarker guidelines (1). In clinical practice, these tests have two roles. They provide a first-line entry point for Lynch syndrome (LS) screening and guide subsequent germline testing and family-based genetic management (2). They also help predict benefit from immune checkpoint inhibitors and influence treatment selection in advanced or locally advanced disease (3, 4).

Routine use of deficient mismatch repair/microsatellite instability-high (dMMR/MSI-H) and mismatch repair-proficient/microsatellite-stable (pMMR/MSS) categories has linked these results to treatment and screening pathways. The distinction may be overextended when a tumor-level functional phenotype is used to infer germline risk. In the classical two-hit model, Lynch syndrome-associated tumors arise when a germline pathogenic MMR variant is followed by a somatic second hit, such as loss of heterozygosity, somatic mutation or promoter methylation. This process can lead to loss of MMR function and an MSI-H phenotype. However, some tumors in carriers of germline MMR variants may not acquire a second hit in the corresponding MMR gene. They may instead develop through other molecular routes, including chromosomal instability, while retaining proficient MMR function. This produces separation between genotype and tumor phenotype. Ranganathan et al. reported that approximately 10% of colorectal cancers in patients with Lynch syndrome were pMMR, with higher proportions among PMS2 and MSH6 carriers (5). Studies of assay concordance also described discordance between MMR IHC, MSI assays and sequencing-based MSI assessment. Tumor heterogeneity, specimen quality, testing platforms and interpretive thresholds may contribute to these differences (6–8). These findings limit the use of tumor phenotype as a substitute for germline genetic testing.

This separation between phenotype and genotype may be difficult to recognize in PMS2-associated Lynch syndrome. Pathogenic PMS2 variants have relatively low penetrance, and family histories may be uninformative. Interpretation is also complicated by highly homologous regions in the PMS2CL pseudogene. When hereditary evaluation stops solely because the tumour is pMMR/MSS, a germline variant and associated familial risk may be missed.

We report a patient with early-onset low rectal adenocarcinoma whose tumour showed a pMMR/MSS phenotype, whereas subsequent PMS2-specific testing of peripheral-blood DNA confirmed a germline pathogenic PMS2 variant. The case is used to discuss why pMMR/MSS should not be interpreted in isolation in patients with early-onset disease or other high-risk features, and how tumour treatment stratification should be separated from hereditary risk management.

## Materials and methods

### Clinical data

This retrospective single-case analysis used records from the First Hospital of Jilin University and the patient's previous medical documentation. We reviewed endoscopy, imaging, surgical pathology, immunohistochemistry, paired tumor-peripheral blood sequencing, circulating tumor DNA (ctDNA) molecular residual disease (MRD) testing and follow-up data. Clinical data were de-identified during manuscript preparation. The patient provided written informed consent for publication. The study was approved by the Ethics Committee of the First Hospital of Jilin University (approval number: 23K162-002).

### MMR immunohistochemistry

MMR IHC was performed on a Leica Bond-III automated immunohistochemistry staining system (Leica, Deer Park, IL) with MLH1 (clone BC23), PMS2 (clone A16-4), MSH2 (clone FE11), and MSH6 (clone BC19) antibodies (Biocare Medical, Pacheco, CA). Staining was performed according to the kit instructions and departmental procedures. Interpretation followed the ASCP/CAP molecular biomarker guideline for colorectal cancer (1). Positive nuclear staining in stromal lymphocytes and normal intestinal epithelial cells served as internal controls. Two senior pathologists reviewed the slides independently, and disagreements were resolved by consensus.

### High-throughput sequencing of tumor tissue and peripheral blood

Targeted next-generation sequencing (NGS) of tumour tissue and plasma circulating tumour DNA (ctDNA) was performed on the Illumina HiSeq 4000 platform. Blood samples were collected on 17 September 2025, approximately three weeks after curative surgery.

Tumor tissue and matched peripheral-blood leukocyte DNA were subjected to genomic profiling for somatic variant detection and germline hereditary cancer risk assessment, respectively. Tumor tissue and paired leukocyte control DNA were analysed using a 437-gene somatic tumor panel, with a mean sequencing depth of 1287.37  ×  and an effective depth of 1117.41  × . Independent germline predisposition testing was performed on leukocyte-derived DNA using a 125-gene hereditary tumour panel. Somatic variant filtering was conducted using matched white blood cell (buffy coat) DNA to exclude constitutional variants and clonal haematopoietic mutations via an automated analytical pipeline (ATG-Seq).

For postoperative molecular residual disease (MRD) assessment, plasma ctDNA was analysed using the Shielding™ Ultra 2365-gene panel by ultra-deep sequencing. The mean sequencing depth was 40,846.22  × , and the effective sequencing depth was 6,991.38  × . MRD positivity was defined as the detection of at least one tumour-associated somatic variant in plasma that was concordant with the primary tumour profile.

MSI status was determined purely from tumour sequencing data based on 61 microsatellite loci and MMR-associated genes covered by the panel. Tumour mutational burden (TMB) was defined as the number of non-synonymous somatic variants per megabase of coding sequence. Detailed sequencing parameters are summarised in Table 1.

**Table 1:** Molecular findings in tumour tissue and matched peripheral blood/plasma from a patient with an analytically confirmed germline pathogenic PMS2 variant.

Item	Rectal cancer tissue sample (1118385118-F)	Peripheral blood/ctDNA sample (1118385118-P)
NGS testing date	Sample collection: September 17, 2025; report: September 30, 2025	Sample collection: September 17, 2025; report: September 30, 2025
Sample source	FFPE scrolls from the rectal tumor surgical specimen	Whole blood; leukocyte DNA was used for matched-control/germline assessment, and plasma derived from the same collection was used for ctDNA MRD assessment
Clinical diagnosis	Rectal cancer	Matched peripheral blood sample
Testing scope	437-gene tumor tissue panel, approximately 1.53 Mb; covering 61 microsatellite loci and MMR-related genes	2365-gene customized MRD panel; 125 hereditary tumor-predisposition genes
MMR IHC pattern	MMRp	Not applicable
MSI status	MSS	Not applicable
TMB	TMB-L, 7.21 mutations/Mb	Not applicable
Tumor cell content/ sample QC	Tumor cell content acceptable (>=20%); mean sequencing depth 1287.37x; effective sequencing depth 1117.41x; overall quality acceptable	Mean sequencing depth 40846.22x; effective sequencing depth 6991.38x; overall quality acceptable
Germline control	Matched peripheral blood	Whole blood
Detected variants		
Germline MMR gene variant	Tumour-tissue NGS identified NM_000535.7(PMS2):c.1577del (p.Asp526AlafsTer69), GRCh37/hg19 chr7:6026819delT, at a VAF of 40.12% (DP = 825; AD = 331).	The clinical laboratory classified NM_000535.7(PMS2):c.1577del as a heterozygous pathogenic germline finding according to its ACMG/AMP-based interpretation; Quantitative germline VAF could not be calculated from the supplied P-sample assay because the locus had no reportable coverage.
Major tumor-specific somatic variants	TP53 c.880del (p.E294Sfs*51), VAF 25.93%; APC c.646C > T (p.R216*), VAF 25.72%; CDC73 c.664C > T (p.R222*), VAF 12.15%; AMER1 c.2449G > A (p.D817N), VAF 18.78%	TP53 c.880del, VAF 0.06%; APC c.646C > T, VAF 0.10%; CDC73 and AMER1 were not detected or not reported in plasma
MRD status	Used to define tumor-specific variants	MRD-positive; low-frequency tumor-specific variants detected in plasma
RAS/BRAF status	No KRAS/NRAS exon 2/3/4 mutations detected; no BRAF V600E mutation detected	Not applicable
PMS2-specific validation	Not applicable	PMS2-specific nested genomic PCR with TA-clone Sanger sequencing of peripheral-blood DNA confirmed PMS2-specific assignment.
PMS2 somatic second-hit assessment	No dedicated analyses of tumoural PMS2 somatic second-hit events, LOH, copy-number alterations or promoter methylation were performed.	Not applicable
Reference genome	GRCh37/hg19	GRCh37/hg19

IHC, NGS, MSI, TMB and ctDNA MRD results are summarized from clinical pathology records and molecular testing reports. MSI status was assessed only in tumour tissue; ctDNA samples were not used for MSI interpretation. Germline variants refer to hereditary variants detected in peripheral blood. Somatic variants refer to tumour-specific variants identified mainly in tumour tissue, not classified as germline, and potentially used for MRD tracking. The clinical laboratory reported NM_000535.7(PMS2):c.1577del (p.Asp526AlafsTer69) as a heterozygous pathogenic germline variant according to its ACMG/AMP-based interpretation. The reported VAF of 40.12% (DP = 825; AD = 331) was derived from the F tumour-tissue sample. The matched P-sample data had no reportable coverage at this locus; therefore, a numerical germline VAF could not be calculated from the available NGS data. PMS2-specific three-step nested genomic PCR of peripheral-blood DNA, followed by bidirectional Sanger sequencing and TA cloning with individual-clone sequencing, supported assignment of the variant to PMS2 rather than PMS2CL and supported its germline interpretation. The variant is listed in ClinVar as pathogenic (Variation ID 1916358; dbSNP rs2535768807).

IHC, immunohistochemistry; NGS, next-generation sequencing; MMR, mismatch repair; MSI, microsatellite instability; MSS, microsatellite stable; TMB, tumor mutational burden; ctDNA, circulating tumor DNA; MRD, molecular residual disease; VAF, variant allele frequency; LOH, loss of heterozygosity.

### PMS2-specific orthogonal confirmation

To exclude amplification of the highly homologous PMS2CL pseudogene, peripheral-blood genomic DNA was analysed using a three-step nested genomic PCR assay designed from sequence differences between PMS2 and PMS2CL. The first- and second-round forward primers were located outside the C-terminal PMS2/PMS2CL homologous region, whereas the third-round PMS2-F primer targeted a PMS2/PMS2CL-discriminating sequence within this region. The reverse primer was located downstream of c.1577del. The first, second and third PCR products were expected to be approximately 3,600 bp, 2,788 bp and 2,385 bp, respectively. The first round used peripheral-blood genomic DNA as template; the second and third rounds used the preceding nested PCR product as template. Each reaction had a final volume of 20 μL and contained 0.5 μL of each 10 μM primer stock, yielding a final concentration of 0.25 μM per primer. The primer sequences were PMS2-nested 1-F (5′-GCCTTAGAATGCGTTGATATC-3′) and PMS2-nested 1-R (5′-AGCCACCACACCTAGCCTCA-3′) for the first round, PMS2-nested 2-F (5′-TCAGGCCATCTTCCTGCCTC-3′) and PMS2-nested 2-R (5′-TCCAGGACAAATCTTTGCCC-3′) for the second round, and PMS2-F (5′-TGCTGGGATTTCCTGACTTCTA-3′) with PMS2-nested 2-R for the third round. PCR products were resolved on a 2% agarose gel, and the expected final band was excised and purified. The final amplicon was sequenced by Sanger sequencing in both directions. (Sequencing platform: Kuntairui (Wuhan) Biotechnology Co., Ltd.) Direct sequencing showed mixed peaks from the deletion site onward. The purified amplicon was therefore TA-cloned, and individual clones were sequenced to distinguish the two alleles. Detailed sequencing information are summarised in Supplementary Tables S1–S2.

### MSI polymerase chain reaction testing

MSI polymerase chain reaction (PCR) testing was performed using the Promega LMR MSI PCR kit (Promega, Madison, WI, USA) according to the manufacturer's instructions. The assay evaluates fragment-length shifts at eight microsatellite markers: BAT-25, BAT-26, NR-21, MONO-27, BAT-52, BAT-56, BAT-59 and BAT-60. Results were classified as MSI-high (MSI-H) when at least two markers were unstable, MSI-low (MSI-L) when one marker was unstable, and microsatellite stable (MSS) when all markers were stable (9).

## Case description

A 41-year-old woman presented with increased stool frequency and intermittent hematochezia for more than 3 months. She reported that her maternal uncle had intestinal cancer, although the pathological type and age at diagnosis were unknown. Baseline colonoscopic biopsy showed low rectal adenocarcinoma with retained MLH1, PMS2, MSH2 and MSH6 expression. Pelvic MRI staged the tumor as cT3N1bM0, with positive mesorectal fascia (MRF) and negative extramural vascular invasion (EMVI). The clinical stage was IIIB. The main clinical course is shown in Figure 1A.

**Figure 1:**
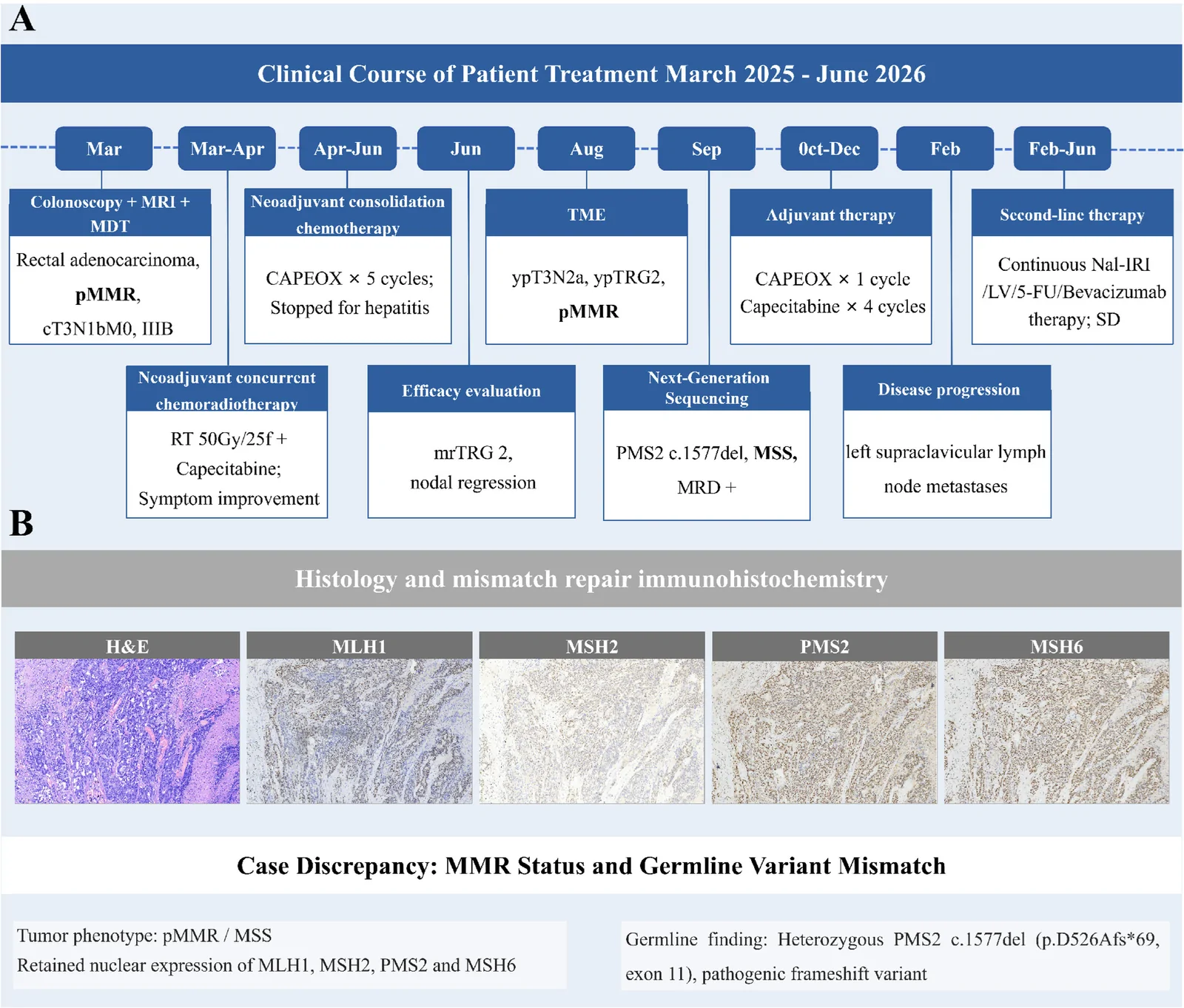
Clinical course and pathological immunohistochemistry findings. **(A)** Timeline illustrating the diagnostic work-up, multimodal neoadjuvant-to-surgical treatment, adjuvant chemotherapy, molecular profiling, disease recurrence, and subsequent second-line clinical-trial-based therapy for the patient from March 2025 to June 2026. **(B)** H&E staining and MLH1, MSH2, PMS2 and MSH6 immunohistochemistry show retained nuclear expression in tumour cells.

The patient received neoadjuvant long-course concurrent chemoradiotherapy followed by five cycles of oxaliplatin plus capecitabine (CAPEOX). Treatment was stopped because transaminase levels increased. Response assessment showed magnetic resonance tumour regression grade (mrTRG) 2, with smaller regional and lateral lymph nodes after treatment. Total mesorectal excision was then performed. Postoperative pathology showed moderately to poorly differentiated rectal adenocarcinoma with tumour regression grade (TRG) 2. The tumour extended beyond the muscularis propria into perirectal connective tissue and involved the dentate line. Lymphovascular and perineural invasion were present. The circumferential resection margin was close to tumour. Four of 20 lymph nodes contained metastases, giving a pathological stage of ypT3N2a. The resection specimen again showed retained expression of all four MMR proteins. The IHC findings are shown in Figure 1B. Paired tumour-peripheral blood sequencing showed that the tumour was MSS, with a TMB of 7.21 mutations/Mb (TMB-L). No KRAS/NRAS exon 2–4 mutations or BRAF V600E mutation were detected. Tumour-tissue NGS identified NM_000535.7(PMS2):c.1577del (p.Asp526AlafsTer69; GRCh37/hg19 chr7:6026819delT) at a variant allele frequency (VAF) of 40.12% (DP = 825; AD = 331). The clinical laboratory classified this variant as a heterozygous pathogenic germline variant based on ACMG/AMP criteria (10). However, the supplied P-sample raw data did not provide reportable coverage at this locus, and therefore a quantitative germline VAF could not be independently calculated. No dedicated analyses of PMS2 somatic second-hit events, loss of heterozygosity, copy-number alterations or promoter methylation were performed in the tumour tissue; therefore, the available data did not establish biallelic PMS2 inactivation. Postoperative ctDNA MRD testing was positive. The molecular findings are summarized in Table 1.

On 16 October 2025, the patient received one postoperative cycle of CAPEOX. Oxaliplatin hypersensitivity occurred during treatment, after which she received oral capecitabine for four additional cycles. These treatment decisions considered the positive MRD result together with the pathological high-risk features, rather than MRD positivity alone. On 11 February 2026, PET/CT showed a left supraclavicular lymph node with high FDG uptake. A subsequent needle biopsy of this lymph node demonstrated metastatic adenocarcinoma. Immunohistochemistry showed CK7 negativity, CK20 positivity, CDX2 positivity and SATB2 positivity, with negative TTF-1, PAX8, GATA3 and ER, consistent with gastrointestinal/colorectal origin and compatible with metastasis from the known rectal primary. Treatment was subsequently switched to a second-line clinical-trial-based regimen consisting of nanoliposomal irinotecan (nal-IRI), leucovorin (LV), 5-fluorouracil (5-FU), and bevacizumab (Bev), for a total of eight cycles, which had been completed by the date of last follow-up. The latest follow-up assessment on 13 June 2026 showed stable disease with shrinkage of target lesions that did not meet criteria for partial response.

To address potential PMS2CL interference in the initial NGS finding, PMS2-specific nested genomic PCR followed by Sanger sequencing and TA-clone sequencing was performed using peripheral-blood DNA. Direct bidirectional Sanger sequencing of the final amplicon showed mixed peaks from the deletion site onward. After TA cloning of the purified amplicon, individual clones yielded either a wild-type sequence or a sequence carrying c.1577del. These results confirmed assignment of the deletion to the PMS2-specific amplicon and supported its heterozygous germline interpretation. Representative electrophoresis and sequencing results are shown in Supplementary Figure S1.

## Discussion

This case concerns a common decision point in hereditary risk screening for colorectal cancer. Once tumor tissue shows retained MMR protein expression (pMMR) and microsatellite stability (MSS), Lynch syndrome evaluation may be stopped and germline testing may not be pursued. This practice follows the classical two-hit model of Lynch-associated carcinogenesis. Its applicability is limited, however, particularly in PMS2-associated Lynch syndrome, where phenotype-led screening can miss germline disease.

From a biological perspective, coexistence of a pMMR/MSS phenotype and a germline pathogenic PMS2 variant is plausible. Lynch syndrome-associated colorectal cancer does not necessarily follow a single route in which a germline variant is followed by a somatic second hit. Ahadova et al. proposed that some tumors may be initiated by other driver events and that MMR deficiency is not required as the initiating condition for every lesion (11). In the present tumor, APC and TP53 mutations were detected, which are typical driver events in the chromosomal instability pathway. PMS2 somatic second-hit events, loss of heterozygosity and promoter methylation were not specifically investigated in this tumour. Therefore, the available data cannot determine whether these mechanisms contributed to its development. Whether this rectal cancer was directly driven by the germline PMS2 variant remains unclear. Concordant MMR IHC results in the pretreatment biopsy and resection specimen, together with tumor sequencing showing MSS, make a simple false-negative explanation less likely. The findings are more consistent with separation between phenotype and genotype. Germline diagnosis and tumor phenotype therefore need separate interpretation, because each has a different clinical use.

This phenotype-genotype separation has particular clinical relevance in PMS2-associated Lynch syndrome. Compared with MLH1- and MSH2-associated subtypes, pathogenic germline PMS2 variants have lower penetrance and less typical familial aggregation. In this case, the patient had only an uncle with intestinal cancer. The pathological type and age at diagnosis were unknown. The usual clinical triggers for genetic evaluation were therefore weak, increasing the possibility of a missed diagnosis. Ten Broeke et al. and subsequent analyses estimated higher risks of colorectal and endometrial cancer in PMS2 variant carriers than in the general population, supporting structured surveillance and family management (12, 13). PMS2 and the PMS2CL pseudogene also contain highly homologous regions. Paixao et al. and Andini et al. discussed the risk of variant misassignment in these regions and the role of orthogonal methods, including long-range PCR, for confirmation (14, 15). In the present case, PMS2-specific nested genomic PCR followed by TA-clone Sanger sequencing confirmed assignment of the variant to PMS2, addressing the principal analytical concern regarding PMS2CL interference.

This screening blind spot is not limited to PMS2-associated disease. In the broader early-onset colorectal cancer population, a pMMR phenotype is also insufficient to exclude germline hereditary risk. In most cases, pMMR/MSS substantially reduces suspicion for Lynch syndrome, but it should not be used as the sole determinant of whether germline testing is considered. The present patient developed disease at 41 years of age and had a family history of gastrointestinal cancer, placing her in a high-risk early-onset colorectal cancer group. If management had proceeded as sporadic colorectal cancer solely because the tumor was pMMR/MSS, cascade testing and multiorgan surveillance for the family could have been delayed. Pearlman et al. performed tumor MMR testing and multigene germline sequencing in 450 patients with colorectal cancer diagnosed before 50 years of age. They reported pathogenic or likely pathogenic germline variants in 16% of patients. Even among patients with MMR-proficient tumors, 8% carried a pathogenic germline variant, and approximately one-third of mutation-positive patients did not meet traditional genetic testing criteria (16). These data do not support indiscriminate germline testing for every pMMR/MSS colorectal cancer. They do show that negative IHC/MSI results should not be used as the end point of hereditary risk assessment in high-risk patients.

The distinction between phenotype and genotype has practical consequences for clinical decision-making. In treatment selection, germline PMS2 positivity should not be taken as an indication for immunotherapy. Evidence for immune checkpoint inhibitor benefit is based on the tumor-level dMMR/MSI-H functional phenotype. Cercek et al. also used tumor-level mismatch repair deficiency as the basis for eligibility and response assessment in nonoperative management of dMMR solid tumors (17). In this case, the tumor was pMMR/MSS and TMB-L. Without additional evidence supporting dMMR/MSI-H, immunotherapy based solely on a germline PMS2 variant would have limited evidential support. Treatment should follow principles used for sporadic pMMR colorectal cancer. For prognostic assessment, postoperative ctDNA MRD positivity together with pathological high-risk features indicates increased recurrence risk and may inform the intensity of adjuvant therapy and follow-up (18, 19). This marker is used for recurrence risk stratification of the current tumor. It is not a marker of Lynch syndrome background and does not change the lesion's MMR/MSI classification. If the patient later develops a new colorectal lesion or another Lynch syndrome-associated tumor, MMR/MSI status should be retested in the new lesion. Carlin et al. reported that synchronous and metachronous colorectal tumors may differ in MMR status (20).

Several limitations should be acknowledged. First, although PMS2-specific nested genomic PCR with TA-clone Sanger sequencing confirmed assignment of the germline variant to PMS2, no dedicated analyses of tumoural PMS2 somatic second-hit events, LOH, copy-number alterations or promoter methylation were performed. The causal relationship between the germline variant and this pMMR/MSS rectal carcinoma therefore remains uncertain. Second, the single-patient design prevents estimation of frequency or generalizability. Third, the family history was limited and incompletely verified, and no cascade-testing or segregation results were available. Fourth, the P-sample assay had no reportable coverage at the variant locus, so a quantitative germline VAF could not be calculated; the 40.12% value was the F tumour-tissue VAF. Fifth, postoperative ctDNA MRD was interpreted together with pathological risk factors and should not be regarded as a stand-alone determinant of treatment. Sixth, treatment-related effects on the resection specimen cannot be completely excluded, although the pretreatment biopsy showed retained MMR protein expression. Seventh, follow-up remains relatively short. Finally, this case does not establish that all patients with pMMR/MSS colorectal cancer require germline testing; testing decisions should be linked to age at diagnosis, personal history, family history, tumour features and established hereditary cancer testing criteria.

## Conclusion

This case describes an early-onset pMMR/MSS low rectal adenocarcinoma in a patient with an analytically confirmed germline pathogenic PMS2 frameshift variant. The case illustrates that pMMR/MSS results do not invariably exclude hereditary cancer predisposition in selected early-onset or otherwise high-risk patients. However, because no dedicated analyses of tumoural PMS2 s-hit, loss of heterozygosity, copy-number alterations or promoter methylation were performed, the carcinoma cannot be attributed definitively to PMS2-associated Lynch syndrome. Germline testing should be considered according to age at diagnosis, personal and family history, tumour features and established testing criteria, rather than universally for all pMMR/MSS colorectal cancers. Treatment should be guided by the tumour-level MMR/MSI phenotype, disease stage and recurrence risk.

## Patient perspective

At 41 years old, I was diagnosed with low rectal adenocarcinoma after three months of increased stool frequency and intermittent rectal bleeding. The diagnosis was deeply unsettling; I had no clear, detailed family history of cancer and had not expected to face a serious illness at my age. I underwent neoadjuvant long-course concurrent chemoradiotherapy followed by five cycles of CAPEOX chemotherapy. Treatment was physically challenging—I experienced persistent fatigue, and elevated liver enzymes eventually forced us to pause chemotherapy earlier than planned. I then had total mesorectal excision surgery, which required a lengthy recovery and lasting adjustments to my bowel function.

When genetic testing after surgery identified a germline pathogenic *PMS2* frameshift variant, it brought a new wave of worry. Beyond concerns about my own cancer recurrence, I was anxious about the hereditary cancer risk for my close relatives. Learning that my tumor was microsatellite stable despite the germline mutation was confusing at first, but my care team helped me understand how this difference shapes my treatment plan and long-term surveillance. When cervical lymph node metastasis was detected six months after surgery, it was a major setback. I have since started a new chemotherapy regimen with bevacizumab, and my disease has remained stable to date. I chose to share my case to highlight that young patients with rectal cancer may still need germline genetic evaluation even when standard tumor tests do not suggest a hereditary syndrome, in the hope of helping other patients in similar situations.

## Data Availability

De-identified clinical data supporting this case report are available from the corresponding author upon reasonable request. Original medical records and raw sequencing data are not publicly available because they contain potentially identifiable medical information.
