# Supplementary material for: Early-onset low rectal adenocarcinoma with a pMMR/MSS phenotype in a patient with an analytically confirmed germline pathogenic PMS2 variant: a case report
Source: Front Med (Lausanne). 2026 Oct 30;13:1945282. doi: 10.3389/fmed.2026.1945282 (PMC13759357; doi:10.3389/fmed.2026.1945282)
Supplement: Supplementary file 1 [file Supplementaryfile1.docx]

**Supplementary Materials**

Supplementary materials are intended to document the orthogonal PMS2 assay, sequencing interpretation boundaries and de-identified pathology evidence.

**Supplementary Section S1. PMS2-specific nested genomic PCR and TA-clone Sanger confirmation**

Peripheral-blood genomic DNA was tested using a three-step nested genomic PCR assay designed from sequence differences between PMS2 and PMS2CL. The first- and second-round forward primers were located outside the C-terminal PMS2/PMS2CL homologous region, whereas the third-round PMS2-F primer targeted a PMS2/PMS2CL-discriminating sequence within this region. The first, second and third expected amplicons were approximately 3,600 bp, 2,788 bp and 2,385 bp. The first round used peripheral-blood genomic DNA; the second and third rounds used the preceding nested PCR product. Each 20 μL reaction contained 0.5 μL of each 10 μM primer stock, yielding a final primer concentration of 0.25 μM per primer. The final amplicon was resolved on a 2% agarose gel, purified and sequenced by Sanger sequencing in both directions. (Sequencing platform: Kuntairui (Wuhan) Biotechnology Co., Ltd.) Direct sequencing showed mixed peaks from the deletion site onward. The purified amplicon was then TA-cloned and individual clones were sequenced, yielding wild-type and c.1577del sequence classes.

**Supplementary Figure S1. PMS2-specific orthogonal confirmation. The final approximately 2,385-bp amplicon was subjected to bidirectional Sanger sequencing, which showed mixed peaks from the c.1577del site onward. TA-clone sequencing resolved separate wild-type and c.1577del sequence classes, supporting assignment of the variant to PMS2 rather than PMS2CL.**

**Supplementary Table S1. Nested PCR primers and reaction design**

| **Round** | **Primer** | **Sequence (5′–3′)** | **Template** | **Expected product** |
| --- | --- | --- | --- | --- |
| 1 | PMS2-nested 1-F | GCCTTAGAATGCGTTGATATC | Peripheral-blood gDNA | ~3,600 bp |
| 1 | PMS2-nested 1-R | AGCCACCACACCTAGCCTCA | Peripheral-blood gDNA | ~3,600 bp |
| 2 | PMS2-nested 2-F | TCAGGCCATCTTCCTGCCTC | Nested PCR product 1 | ~2,788 bp |
| 2 | PMS2-nested 2-R | TCCAGGACAAATCTTTGCCC | Nested PCR product 1 | ~2,788 bp |
| 3 | PMS2-F | TGCTGGGATTTCCTGACTTCTA | Nested PCR product 2 | ~2,385 bp |
| 3 | PMS2-nested 2-R | TCCAGGACAAATCTTTGCCC | Nested PCR product 2 | ~2,385 bp |

*Primer stock concentration: 10 μM. Volume per primer: 0.5 μL in a 20 μL reaction. Final concentration: 0.25 μM per primer.*

**Supplementary Table S2. In-silico primer mapping to GRCh37/hg19 PMS2**

| **Primer** | **Sequence (5′–3′)** | **GRCh37/hg19 location** | **Strand** | **Predicted role** |
| --- | --- | --- | --- | --- |
| PMS2-nested 1-F | GCCTTAGAATGCGTTGATATC | chr7:6029573–6029593 | minus | PMS2-specific upstream primer |
| PMS2-nested 1-R | AGCCACCACACCTAGCCTCA | chr7:6025994–6026013 | plus | Downstream primer |
| PMS2-nested 2-F | TCAGGCCATCTTCCTGCCTC | chr7:6029199–6029218 | minus | PMS2-specific nested upstream primer |
| PMS2-nested 2-R | TCCAGGACAAATCTTTGCCC | chr7:6026431–6026450 | plus | Downstream primer |
| PMS2-F | TGCTGGGATTTCCTGACTTCTA | chr7:6028794–6028815 | minus | PMS2/PMS2CL-discriminating final upstream primer |
| Variant | c.1577del | chr7:6026819delT (standard GRCh37/hg19 genomic coordinate) | Reference plus-strand coordinate; pms2.dna stored in reverse-complement orientation | Included within each amplicon |

**Note:** The company-provided pms2.dna sequence was stored in reverse-complement orientation. Primer sequences were aligned in silico to this supplied sequence, and the resulting positions were converted by the authors to the standard GRCh37/hg19 reference-plus-strand coordinate system. Accordingly, the coordinates below were derived by the authors and were not directly reported by the testing company. The first- and second-round forward primers mapped to regions outside the C-terminal PMS2/PMS2CL homologous region, whereas the third-round PMS2-F primer mapped to a PMS2/PMS2CL-discriminating sequence. The predicted genomic spans of the first-, second- and third-round products were chr7:6025994–6029593 (3,600 bp), chr7:6026431–6029218 (2,788 bp) and chr7:6026431–6028815 (2,385 bp), respectively. The variant coordinate, GRCh37/hg19 chr7:6026819delT, lies within all three intervals. In the primer table, “plus” and “minus” denote orientation relative to the GRCh37/hg19 reference plus strand.

**Supplementary Section S2. De-identified pathology documentation**

The de-identified Chinese pathology report and an English translation in the original report layout are shown in Supplementary Fig. S2. Editable source images and documents are supplied as separate supplementary files. The report documents a left supraclavicular-region lymph-node biopsy with adenocarcinoma and an immunophenotype compatible with lower gastrointestinal origin.

**Supplementary Figure S2.** **De-identified pathology documentation. Panel A shows the de-identified Chinese original-format report; panel B shows an author-prepared English translation in the original report layout for reference. It is not an official institutional translation. Patient name, ID/case/hospital numbers, barcodes, QR codes, signatures and contact details were removed.**
